# The influence of social support and sentence length on meaning in life among incarcerated women in Ecuador

**DOI:** 10.3389/fpsyg.2026.1750267

**Published:** 2026-02-25

**Authors:** Livia I. Andrade, Marlon Santiago Viñán-Ludeña, Jorge Benitez, Gabriela Armijos, Ruth Maldonado

**Affiliations:** 1 Department of Psychology, Universidad Tecnica Particular de Loja, Loja, Ecuador; 2 Escuela de Ingeniería, Catholic University of the North, Coquimbo, Chile; 3 Departamento de Ciencias de la Computación e Inteligencia Artificial, ETSI Informática y de Telecomunicación, CITIC-UGR, University of Granada, Granada, Spain

**Keywords:** exploratory analysis, incarcerated woman, meaning in life, sentence duration, social support

## Abstract

**Introduction:**

This study examined the influence of social support and sentence length on the sense of meaning in life among incarcerated women in Ecuador.

**Methods:**

This study employed a non-experimental, cross-sectional design with descriptive, correlational, and exploratory components. Data were collected via a convenience sample of 30 women serving final sentences. The Purpose in Life (PIL) test, the MOS Perceived Social Support Questionnaire, and official records on offense type and sentence duration were employed.

**Results and discussion:**

Results indicated that, on average, participants reported moderate levels of meaning in life, with a tendency toward positive scores. Spearman’s correlation revealed a moderate, positive, and statistically significant relationship between perceived social support and meaning in life (r_s_ = 0.40, *p* = 0.030), a finding confirmed through permutation testing (*p* = 0.033). The Firth logistic regression showed a positive but non-significant trend for social support (*β* = 0.64, OR = 1.90, 95% CI [0.89, 4.63]) and a small effect for sentence length (*β* = 0.023, OR = 1.02, 95% CI [0.95, 1.12]), with an area under the ROC curve of 0.67, indicating modest predictive capacity. *Post-hoc* analysis revealed that the study was powered to detect effects of r ≥ 0.49 with 80% confidence. Findings suggest that higher perceived social support is associted with greater meaning in life, highlighting its importance as a potential protective factor for the psychological well-being and rehabilitation of incarcerated women.

## Introduction

The meaning of life is a fundamental construct that influences people’s mental health, defined as the perception of purpose or meaning a person attributes to their existence. This concept was introduced by Frankl (1946), who suggested that the meaning of life emerges when individuals search for their life’s purpose, allowing them to establish clear objectives and goals. This ongoing quest for meaning fosters maturity, personal growth, and the fulfillment of the human potential, emphasizing the human capacity to transcend difficulties and overcome various adversities throughout life (Chan Chi and Chan Chi, 2021). Consequently, Crumbaugh and Maholick (1969) developed a questionnaire to measure life’s meaning, called the Purpose in Life Test (PIL).

The literature suggests that the search for a meaning in life is associated with greater emotional, social, and physical well-being. People who feel their life has meaning or purpose tend to experience higher levels of satisfaction and resilience, which can protect them against mental health problems. Conversely, when individuals lack meaning in life, often referred to as existential emptiness, it can lead to negative consequences in various disorders, such as depression, anxiety, and stress (Camargo Barrero et al., 2020; Molina-Coloma et al., 2022; Schnell, 2021) Similarly, other studies indicate that university students with low levels of life meaning show limitations in learning and do not fully develop their abilities, often devaluing them, leading to a negative self-concept (Sevilla-Santo et al., 2021). Additionally, Huamani (2019), concludes that cancer patients with medium to high levels of life meaning exhibit only 15% incidence of neurosis or noogenic depression, thus demonstrating that the meaning of life can be considered a protective factor for mental health.

One of the factors that can affect a person’s sense of meaning in life is the social factor of deprivation of liberty. Deprivation of liberty refers to the restriction of an individual’s right to move freely, imposed by a competent judge through an enforceable sentence, with the penalty being served through various means, such as imprisonment or house arrest (Kajstura and Sawyer, 2024). This situation can have a profound impact on the meaning of life, as it is a context often characterized by violence, hopelessness, hierarchical structures, survival, and isolation. In this regard, studies conducted by Cossetin-Costa et al. (2023) confirm that the most common health issues reported by inmates include respiratory, gastrointestinal, mental, cardiovascular, and musculoskeletal diseases. The conditions faced by women deprived of liberty in Ecuador, according to Almeida (2017) include lack of education, unemployment, family breakdown, and poverty. These factors often characterize the social reality of women who break the law, and the adverse effects of the sentences they serve extend beyond prison walls, impacting their family networks.

Another important factor for the meaning in life is social support. While meaning in life provides direction and purpose, social support—through the networks in one’s context—strengthens this sense of meaning or purpose. Research highlights that perceived social support reinforces both the sense of purpose and overall well-being (González Casas, 2020; Yuan et al., 2024). However, in the absence of social support, the literature indicates negative effects. For instance, more than 50% of women deprived of liberty do not receive visits from relatives, spouses, or partners, and they experience abandonment from their social and family networks (Cabanillas and Escalante, 2020; Yurrebaso et al., 2022), which generates feelings of loneliness and isolation, ultimately affecting their sense of meaning in life.

Another factor of interest in this research is whether the length of the sentence affects the meaning of life, as time in prison can be perceived as an existential loss or stagnation. Researchers such as Molina-Coloma et al. (2022) note that shorter prison terms are associated with a greater presence of clinical patterns such as antisocial, aggressive-sadistic, compulsive, schizotypal, and borderline personality disorders, as well as alcohol and substance dependence, post-traumatic stress disorder, thought disorder, and delusional disorder. This suggests that time in prison influences the mental health of inmates. Additionally, Vargas et al. (2023) concluded that the length of imprisonment, sentence duration, and the presence or absence of a sentence were also associated with suicide risk. Similarly, Rodriguez and Cardozo (2023) pointed out that prisoners sentenced to terms exceeding their life expectancy had a higher risk of suicide compared to those with shorter sentences. On the other hand, other research indicates that 50% of inmates only began thinking about their life plans months before being released, suggesting that prison becomes a turning point for them: “If they had not entered prison, they would not have thought about a project” (Chuquimia, 2024).

Understanding the meaning of life is particularly important for women deprived of liberty, as incarceration presents unique psychological and social challenges that can severely impact their mental health and overall well-being. In prison, the search for meaning can be a crucial coping mechanism, helping individuals navigate the difficulties of confinement, such as isolation, stigma, and the disruption of social and familial ties. For incarcerated women, who often face additional challenges related to motherhood, social expectations, and gender-based stigma, fostering a sense of purpose can significantly influence their emotional resilience and capacity to rebuild their lives (Bahamón, 2024; Parada Lugo, 2025).

Moreover, exploring the meaning in life within this context is essential for promoting social integration and coexistence within prison environments. A heightened sense of purpose has been linked to improved interpersonal relationships, cooperation, and reduced conflicts among inmates, thereby contributing to a more harmonious prison atmosphere (Turney et al., 2023). Furthermore, facilitating personal growth and self-reflection through programs that nurture life purpose can empower women to engage in transformative experiences that enhance their self-esteem, identity reconstruction, and overall psychological adjustment (Comisión Interamericana de Derechos Humanos, 2023; Comisión Nacional de los Derechos Humanos, 2022).

Social support is crucial for the physical and mental well-being of women in prison. The absence of support networks can intensify the negative effects of incarceration, while family and friends significantly contribute to emotional well-being (Rengifo and DeWitt, 2019). Additionally, the type of crime and length of the sentence can shape incarcerated women’s perception of meaning in life. For instance, women convicted of drug-related offenses often face heightened stigma, affecting their self-esteem and hindering their reintegration process.

From a broader perspective, supporting incarcerated women in finding meaning in life also aids in their reintegration into society upon release. Therefore, understanding the factors that influence the meaning of life among this population—such as social support, type of crime, and length of sentence—is crucial for designing effective interventions that promote psychological well-being, personal development, and successful social reintegration. This study aims to contribute to this understanding by examining the complex interactions between these variables, ultimately informing policy and practice within correctional and rehabilitation systems (Comisión Interamericana de Derechos Humanos, 2023; Galván et al., 2006).

Research on meaning in life within prison environments, particularly among women, remains limited, especially in Latin American contexts. Existing studies suggest that psychosocial resources such as social support may play a relevant role in sustaining psychological well-being during incarceration; however, empirical evidence in female prison populations is still scarce and fragmented.

In this context, the present study adopts an exploratory approach to examine the associations between perceived social support, sentence length, and meaning in life among women deprived of liberty in Zone 7, Ecuador. Rather than testing causal hypotheses, the study seeks to identify preliminary patterns and theoretically meaningful relationships that may inform future confirmatory research in this underrepresented population.

Specifically, the study addresses the following exploratory research questions:

(1) Is perceived social support associated with meaning in life among incarcerated women?
(2) Is sentence length related to variations in meaning in life within this population?

Given the exploratory design, the non-probabilistic sampling strategy, and the contextual specificity of the study setting, the findings are not intended to be generalized beyond the sample. Instead, they provide context-sensitive empirical insights that may be relevant for understanding meaning in life among incarcerated women in similar institutional settings and for guiding subsequent research with larger and more diverse samples.

## Methodology

The research is non-experimental in nature, as the variables are not manipulated; it is cross-sectional, descriptive, correlational and exploratory. Convenience sampling, a non-probabilistic technique, is used because individuals are selected based on specific qualities and characteristics, and the sample is readily accessible to the researcher (Hernández-Sampieri et al., 2014).

### Participants

Initially, a sample of 42 women deprived of liberty with a final sentence was selected, of which 30 agreed to participate in the research project. Psychological tests were administered to those who met the inclusion criteria. However, 12 tests were excluded due to missing signed informed consent forms, incomplete responses, or responses filled out at random. The final sample consists of 30 women deprived of liberty, most of whom (46.7%) are aged between 18 and 28 years and are single (53.3%). The educational level of 33.3% of the participants corresponds to the Unified General Baccalaureate. Additionally, 76.7% reside in urban areas. The socioeconomic level of 53.3% of the women is classified as low. The sociodemographic data are detailed in Table 1.

**Table 1:** Sociodemographic results.

Sociodemographic variable	Level	N(%)
n		30 (100%)
Age (%)	1 (18 a 28)	14 (46.7)
	2 (29 a 39)	8 (26.7)
	3 (> = 40)	8 (26.7)
Gender (%)	1 (women)	30 (100.0)
Marital status (%)	1 (single)	16 (53.3)
	2 (married)	3 (10.0)
	3 (separated)	2 (6.7)
	4 (common-law union)	8 (26.7)
	5 (other)	1 (3.3)
Study level (%)	1 (Pre-school)	1 (3.3)
	2 (Elemental school)	2 (6.7)
	3 (Media school)	9 (30.0)
	4 (Superior school)	4 (13.3)
	5 (High school)	10 (33.3)
	6 (College)	4 (13.3)
Housing sector (%)	1 (Urban)	23 (76.7)
	2 (rural)	7 (23.3)
Socioeconomic level (%)	1 (High)	0
	2 (Medium)	14 (46.7)
	3 (Low)	16 (53.3)

### Instruments

The following instruments were used to carry out the study:

a) *Psychosocial Factors Questionnaire:* The psychosocial-demographic variables assess general characteristics related to social support, family structure, and socioeconomic level. The questionnaire is divided into two parts. The first part consists of 9 items with brief response options and includes the following variables: age, gender, marital status, number of children, educational level, sector of residence, socioeconomic level, sources of income, and life satisfaction. In the second part, the MOS Perceived Social Support Questionnaire (Sherbourne and Stewart, 1991) was used. It consists of 20 items related to social support, with responses on a Likert scale. The first item assesses the size of the social network, while the remaining items form 4 scales that measure emotional support, material or instrumental help, social relationships for leisure and distraction, and affective support, which refers to expressions of love and affection. Normative data indicate that the maximum global social support index is 94, with a mean of 57 and a minimum of 19. The higher the score, the greater the perceived social support. The questionnaire has been validated in Spain, Colombia, and Peru and has demonstrated a high level of reliability (Londoño Arredondo, 2012).
b) *The Purpose in Life Test (PIL)* by Crumbaugh and Maholick (1969): measures the meaning of life versus existential emptiness from a logotherapeutic perspective. The test demonstrates a high reliability coefficient (0.84) and has been empirically and scientifically validated in Spanish, Colombian, Mexican, Cuban, and other populations, with its internal consistency tested, achieving a Cronbach’s alpha of 0.88 (Noblejas et al., 1994; Noblejas de la Flor, 2000).

The test consists of three parts:

◦Part A includes 20 items where the participant rates their feelings on a scale of 1 to 7 between two extreme statements (Likert-type scale).
◦Parts B and C provide additional information for clinical interpretation. In Part B, the participant completes a general sentence about the meaning of their life and freely describes their life situation. In Part C, they detail their purposes, goals, ambitions, and their progress toward achieving them.
  Interpretation is primarily based on the scores from Part A:

- Level 1: Scores below 91 indicate a lack of meaning in life.
- Level 2: Scores between 92 and 112 represent an intermediate or unclear zone regarding the meaning of life.
- Level 3: Scores above 113 indicate the presence of meaning in life.

### Procedure

Data collection was conducted in several phases. First, we approached the directors of the Mixed Social Rehabilitation Center of Loja to introduce and explain the research project. This was followed by a meeting and the signing of an authorization letter, granting the research team access to the center. The center provided a list of individuals with final sentences. The project was then presented to the women deprived of liberty serving final sentences in the “Santa Martha” Pavilion. Voluntary informed consent was obtained, emphasizing the anonymous and confidential nature of the information provided. The psychological instruments were explained to the participants to ensure they followed the guidelines during their completion. Finally, the psychological tests were administered to those who met the inclusion criteria. Testing was conducted in groups of six, in designated rooms within the rehabilitation center, to avoid overcrowding and other potential issues. For data analysis, the R software was used to perform descriptive analyses. Measures of central tendency (arithmetic mean, mode) and standard deviation were calculated for the general scores of each psychological instrument to assess the variables of meaning in life and social support.

### Sampling and generalizability

The present study used a convenience sample of women with final sentences who were available and willing to participate at the Mixed Social Rehabilitation Center of Loja. We acknowledge that convenience sampling does not provide a probability-based estimate of the target population and thus limits generalizability. To partially address this limitation, we (a) compared key sociodemographic characteristics of our sample (age groups, offense type, urban/rural residence, educational level) with publicly available administrative statistics for Ecuadorian prisons and (b) used analytic methods (Firth logistic regression, bootstrap confidence intervals, and permutation tests) that reduce small-sample bias and present effect sizes and uncertainty explicitly.

### Data analysis

Data analysis was conducted following a structured, objective-driven analytical strategy designed to account for the exploratory nature of the study and the constraints associated with a small, non-probabilistic sample. All analyses were performed using R software.

The primary outcome variable, meaning in life, was assessed using the Purpose in Life Test (PIL). For inferential analyses, the total PIL score (Part A) was dichotomized into presence of meaning in life (1) versus absence or unclear meaning in life (0), based on established interpretative cut-offs. This operationalization facilitated both categorical association analyses and logistic regression modeling.

Key predictor variables included perceived social support (continuous total score from the MOS Social Support Survey), sentence length (continuous, measured in years), type of crime (categorical), and age group (categorical). Prior to inferential analyses, data were examined for missing values, distributional properties, and coding consistency.

Descriptive statistics were first computed to characterize the sample. For continuous variables, measures of central tendency (mean, median) and dispersion (standard deviation, interquartile range) were calculated. For categorical variables, absolute and relative frequencies were reported. This step aimed to contextualize the sociodemographic, psychosocial, and legal characteristics of the participants.

Given the exploratory aim of the study, bivariate analyses were conducted to identify potential associations between meaning in life and the explanatory variables. For associations between categorical variables, Cramér’s V was used as a measure of effect size, accompanied by chi-square tests of independence. For associations involving one dichotomous and one continuous variable, point-biserial correlations were computed. The relationship between perceived social support and meaning in life was additionally examined using Spearman’s rank correlation coefficient, due to the non-normal distribution of the variables. These analyses were intended to identify candidate relationships for subsequent multivariable modeling rather than to serve as definitive hypothesis tests.

To assess the robustness of the observed association between perceived social support and meaning in life under small-sample conditions, a permutation-based test (10,000 permutations) was conducted for Spearman’s correlation coefficient. This non-parametric approach avoids reliance on asymptotic assumptions and provides a more reliable *p*-value in the context of limited sample size.

To examine the joint influence of perceived social support and sentence length on meaning in life, logistic regression modeling was performed with meaning in life (binary) as the dependent variable. Prior to model estimation, multicollinearity among predictors was assessed using Variance Inflation Factors (VIF), with all values remaining below the conventional threshold (VIF < 5).

Given the small sample size and the risk of separation bias, a Firth penalized logistic regression was employed. This method provides bias-reduced parameter estimates and is recommended for small-sample and sparse-data contexts. Regression coefficients, odds ratios (OR), and 95% confidence intervals were reported to emphasize effect size and estimation uncertainty.

Model performance was evaluated using the area under the Receiver Operating Characteristic curve (AUC-ROC) as an index of discriminative ability.

To further assess the stability of regression estimates, bootstrap resampling (2,000 replications) was used to generate percentile-based confidence intervals for key regression coefficients. Additionally, age was tested as a potential covariate in extended models; however, its inclusion did not meaningfully improve model fit or discrimination and was therefore excluded from the final model.

A post-hoc power analysis was conducted to contextualize the interpretation of non-significant findings. With a sample size of *n* = 30 and *α* = 0.05, the study was powered to detect correlations of approximately r ≥ 0.49 with 80% power. Accordingly, smaller effects may not have been detectable, and non-significant results should be interpreted cautiously within the exploratory framework of the study.

All statistical tests were two-tailed, and statistical significance was evaluated at *p* < 0.05, while prioritizing effect sizes and confidence intervals to support transparent interpretation.

## Results

Table 1, shows the sociodemographic and contextual characteristics of the participants.

The predominant age group among the participants ranges from 18 to 28 years. The entire sample is female, with 53% being single. Most of the participants have completed basic secondary education (30%) or general high school education (33.3%). Additionally, 76.7% of the women live in urban areas. Regarding socioeconomic status, 53.3% of the participants consider themselves to have a low socioeconomic level, while 46.7% identify as having a medium socioeconomic level. None of the participants consider themselves to be in a high socioeconomic bracket. This distribution generally coincides with national data on the incarceration of women in Ecuador.

The results show that female prisoners, on average, fall into the zone of uncertainty regarding the meaning in life, with a tendency toward higher values. They also report high levels of perceived social support. Most of these women are serving sentences for drug trafficking, which may be related to their low economic status. Future research may help determine whether the presence of a sense of purpose among prisoners is a contributing factor to Loja being considered the least violent prison in the country.

Associations between meaning in life and categorical variables were assessed using Cramér’s V. A moderate association was observed between age group and meaning in life (Cramér’s V = 0.16), although this association was not statistically significant (χ^2^ test, *p* = 0.68). A moderate association was also observed between meaning in life and perceived social support (Cramér’s V = 0.36), as well as between meaning in life and type of crime (Cramér’s V = 0.36); however, neither association reached statistical significance (*p* = 0.126 and *p* = 0.83, respectively).

The point-biserial correlation between meaning in life and sentence length was weak and negative (*r* = −0.25, *p* = 0.23), indicating that longer sentences tended to be associated with lower meaning in life scores, although this relationship was not statistically significant.

These analyses were considered exploratory and served to identify candidate relationships for subsequent robust and multivariable analyses.

Given the theoretical centrality of social support, its association with meaning in life was examined in greater depth. Spearman’s rank correlation coefficient revealed a moderate, positive, and statistically significant association between perceived social support and meaning in life (r_s_ = 0.40, *p* = 0.030), indicating that participants who reported higher levels of social support also tended to report a stronger sense of meaning in life.

To assess the robustness of this finding under small-sample conditions, a permutation-based test was conducted. The permutation test confirmed the statistical significance of the association (*p* = 0.033), suggesting that the observed relationship was unlikely to have occurred by chance despite the limited sample size.

To examine the joint influence of perceived social support and sentence length on meaning in life, logistic regression models were estimated with meaning in life (high vs. medium/low) as the dependent variable.

Initial models were evaluated for multicollinearity, with all predictors showing acceptable Variance Inflation Factor values (VIF < 5). The final model, selected through bidirectional stepwise selection using AIC, included social support and time of condemnation. The model demonstrated good discriminative ability with an AUC-ROC of 0.83. Overall classification accuracy was 76.67%, with sensitivity of 57.14% and specificity of 82.61%. Table 2 shows a summary about logistic regression analysis. Despite the results are not statistically significant due to the lower number of observation (*n* = 30) this shows the possible relation between meaning in life and social support and time of condemnation.

**Table 2:** Logistic regression analysis.

Independent variables	β (SE)	Odds ratio (95% CI)	*p*-value
Social support	2.35 (1.20)	10.44 (1.32–226.49)	0.05
Time of condemnation	0.34 (0.35)	1.41 (1.02–4.13)	0.33

Given the small sample size and the potential for separation bias, a Firth penalized logistic regression was applied.

The model indicated a positive, though non-significant, effect of perceived social support on the likelihood of reporting high meaning in life (*β* = 0.64, OR = 1.90, 95% CI [0.89, 4.63], p=.097 ). Sentence length showed a small positive but non-significant effect (*β* = 0.023, OR = 1.02, 95% CI [0.95, 1.12], p=.54 ). Although the model did not reach statistical significance overall, the direction of effects was consistent with theoretical expectations.

Model discrimination was modest, with an area under the ROC curve (AUC = 0.67), indicating acceptable but limited predictive capacity, suggesting that higher perceived social support modestly increased the probability of reporting a high sense of meaning in life.

To evaluate the stability of the regression estimates, bootstrap resampling (2,000 replications) was conducted. The percentile-based bootstrap confidence interval for the social support coefficient ranged from −0.11 to 1.80, highlighting estimation uncertainty but reinforcing the positive direction of the association.

Age was examined as a potential covariate in extended models; however, its inclusion did not substantially improve model fit or discrimination (AUC increased marginally from 0.83 to 0.88) and was therefore excluded from the final model.

A post-hoc power analysis indicated that, with a sample size of *n* = 30 and *α* = 0.05, the study had approximately 80% power to detect correlations of r ≥ 0.49. This suggests that the study was adequately powered to detect moderate-to-large effects but had limited sensitivity to smaller associations. Consequently, non-significant findings should be interpreted cautiously within the exploratory framework of the study.

## Discussion

The results indicate that female prisoners, on average, experience uncertainty regarding their meaning in life, although there is a tendency toward positive values. This finding raises important questions about the psychological state of incarcerated women and their ability to find purpose despite adverse circumstances. The high levels of perceived social support suggest that interpersonal relationships play a significant role in maintaining emotional well-being (Eades, 2019). This aligns with existing literature, which emphasizes the importance of social bonds in fostering resilience and psychological stability among incarcerated populations (Folk et al., 2019; Hayes, 2016). However, the nature and sources of this social support—whether from family, fellow inmates, or institutional programs—warrant further exploration to understand how they specifically contribute to life purpose.

Many incarcerated women come from low socioeconomic backgrounds and marginalized communities. These conditions of social vulnerability, often coupled with addiction, are closely linked to criminal activities, including drug trafficking (Eades, 2019; Prison Policy Initiative, (2024)). A notable contextual factor is that most participants are serving sentences for drug trafficking, likely influenced by socioeconomic vulnerabilities (Oficina de las Naciones Unidad contra la Droga y el Delito (UNODC), 2023). This invites reflection on the broader social determinants of crime and how systemic issues, such as poverty and lack of opportunities, may contribute to criminal behavior (Fleetwood, 2014). These findings highlight the need for social policies that address economic disparities and provide support systems for at-risk populations, particularly women. Furthermore, this context raises ethical and philosophical questions about the justice system’s role in rehabilitation versus punishment, especially for crimes driven by socioeconomic necessity.

The moderate association between social support and meaning in life implies that enhancing social networks and support systems within prisons could be a viable intervention strategy. It is essential for incarcerated women to have a space to process their feelings and experiences both during and after their time in prison. A sense of belonging, social capital, and group therapy are valuable tools that provide a supportive environment where women can build genuine relationships and share their experiences without fear of judgment. Mental health professionals can help them find positive meaning in their lives rather than focusing solely on feelings of guilt. These interventions are crucial for empowering women to rebuild their life plans and enhance their sense of meaning and purpose (Yang, 2024). This could involve strengthening family connections, peer support groups, or mentorship programs aimed at fostering personal growth and purpose (Mccrary et al., 2022; Perrin, 2025).

The logistic regression analysis further underscores the potential impact of social support and time of condemnation on meaning in life. Although not statistically significant, the model’s good discriminative ability suggests that these variables play a substantial role in influencing life purpose. Notably, the negative correlation between meaning in life and time of condemnation indicates that longer sentences are associated with lower purpose in life scores. This finding challenges the effectiveness of long-term incarceration as a rehabilitative approach, particularly for non-violent offenses such as drug trafficking. It prompts a critical reevaluation of sentencing policies, suggesting that restorative justice and alternative sentencing programs might better support psychological well-being and social reintegration (Cullen et al., 2011; Taxman and Breno, 2017).

The stark contrast in median time of condemnation between those with high (7.14 years) and low (2.04 years) meaning in life scores warrants further reflection. It suggests that prolonged imprisonment may exacerbate feelings of hopelessness or existential crisis, reinforcing the need for psychological interventions that promote purpose and personal development. These could include therapeutic programs, educational opportunities, and vocational training designed to empower incarcerated women to envision a meaningful future post-incarceration (Crewe et al., 2017; Haney, 2017; Lucía et al., 2024).

In addition, the results revealed a moderate positive association between perceived social support and meaning in life. This indicates that women who perceive higher levels of emotional and instrumental support tend to report a stronger sense of meaning and purpose during incarceration. The direction and strength of this relationship align with prior research suggesting that social support acts as a crucial protective factor for maintaining psychological well-being and existential coherence in adverse conditions (Frankl, 2006; Steger, 2017; Delle Fave and Soosai-Nathan, 2014).

Although the firth logistic regression model did not reach conventional significance levels, the positive trend observed for social support suggests that higher perceived support may increase the likelihood of experiencing high meaning in life. A small association between sentence length and meaning in life was observed, although the underlying mechanisms remain unclear and were not examined in the present study.

The use of firth penalized regression, bootstrap confidence intervals, and permutation testing strengthens the reliability of results in the context of a small sample (*n* = 30). These analyses demonstrate that, even in constrained prison samples, rigorous analytical strategies can yield interpretable and transparent results that respect statistical assumptions and highlight uncertainty.

Importantly, the absence of statistically significant global effects does not imply a lack of practical or theoretical relevance. The observed—modest—relationships are consistent with the international literature emphasizing the centrality of social connectedness, family ties, and perceived support for fostering meaning, resilience, and post-traumatic growth among incarcerated populations (Vanhooren et al., 2018; Jang et al., 2021). In this sense, the current findings should be interpreted as exploratory evidence that underscores the psychological benefits of social integration and affective bonds even in contexts of severe deprivation.

## Conclusion

The present exploratory study examined the relationship between perceived social support, sentence length, and meaning in life among women deprived of liberty in Zone 7, Ecuador. The findings indicate that perceived social support is moderately and positively associated with meaning in life, while sentence length shows a weak and inconsistent relationship with this construct. These results suggest that interpersonal resources may be more relevant to meaning in life during incarceration than legal variables such as sentence duration.

Across multiple analytical approaches, perceived social support consistently showed a positive association with meaning in life, although not all effects reached conventional levels of statistical significance. This pattern underscores the potential importance of social support as a psychosocial correlate of existential meaning, while also highlighting the need for cautious interpretation given the exploratory design and limited statistical power.

In contrast, age, type of crime, and sentence length did not demonstrate robust or statistically significant associations with meaning in life in this sample. While moderate effect sizes were observed in some exploratory analyses, these findings should be interpreted as preliminary and not as evidence of strong or definitive relationships.

Overall, the results contribute preliminary empirical evidence to a population that remains underrepresented in quantitative research. The study highlights perceived social support as a variable warranting further investigation in relation to meaning in life among incarcerated women. Future research should seek to replicate these findings using larger, probabilistic samples and longitudinal designs to clarify the strength, direction, and temporal dynamics of these associations.

## Data Availability

The raw data supporting the conclusions of this article will be made available by the authors, without undue reservation.
